# Prelacteal feeding practice and its associated factors among mothers of children age less than 24 months old in Southern Ethiopia

**DOI:** 10.1186/s13052-019-0604-3

**Published:** 2019-01-15

**Authors:** Esayas Aydiko Amele, Birhanu wondimeneh Demissie, Kalkidan Wondwossen Desta, Emebet Berhane Woldemariam

**Affiliations:** 1 Department of Nursing, College of Medicine and Health Sciences, Wolaita Sodo University, Sodo, Ethiopia; 2 School of Nursing and Midwifery, College of Health Science, Addis Ababa University, Addis Ababa, Ethiopia

**Keywords:** Pre-lacteal feeding, Associated factors, Infants

## Abstract

**Background:**

Although Pre-lacteal feeding is a barrier for implementation of optimal breastfeeding practices and increases the risk of neonatal illness and mortality, still it is continued as a deep-rooted nutritional malpractice in developing countries. In Ethiopia pre-lacteal feeding continued as one of the nutritional malpractices in newborns. Therefore the aim of this study was to assess pre-lacteal feeding practice and its determinants among mothers of children less than 24 months of age in Sodo zuria district, Wolaita zone, Southern Ethiopia.

**Methods:**

Community-based cross-sectional study was conducted from February 15, 2017 to March 12, 2017 in Sodo zuria district, Wolaita zone, Southern Ethiopia. Five hundred five (505) mothers of children aged less than 24 months were selected by multistage randomized sampling technique and the data were collected by using interview based structured questionnaire. Descriptive statistics, binary and multivariable logistic regression analysis were employed to identify the factors associated with pre-lacteal feeding practices. Variables with a *p*-value < 0.05 were identified as statistically significant factors.

**Results:**

The prevalence of pre-lacteal feeding practice was 20.6%. The common type of pre-lacteal feeding given was plain water; 38(7.7%) and the major reason was insufficient breast milk 32(6.5%). Mothers living with extended family type (AOR = 10.64, 95% CI: 1.05, 10.71), Lack of breastfeeding counseling (AOR = 5.16, 95% CI: 1.76, 15.13) and mothers who avoid colostrum (AOR = 9.72, 95% CI: 3.46, 27.30) were statistically significant positive predictors of pre-lacteal feeding practice.

**Conclusion & Recommendation:**

Pre-lacteal feeding is commonly practiced in Soddo zuria district. Mothers who live with extended family type, mothers who did not get breastfeeding counseling and mothers who avoid colostrum were statistically significant positive predictors of pre-lacteal feeding practice. Therefore, strengthening breastfeeding counseling about the risks associated with pre-lacteal feeding and colostrum feeding intervention should be integrated. Promotion of intensive nutrition education program, on the benefit of colostrum by giving special emphasis to extended family mothers should be implemented in the community.

## Background

Prelacteal feeds are foods given to newborns before breastfeeding is established or before breast milk “comes out,” usually on the first day of life [1]. Although Pre-lacteal feeding is a barrier for implementation of exclusive breastfeeding practices and increases the risk of neonatal illness and mortality, it is continued as a deep-rooted nutritional malpractice in developing countries [2].

Exclusive breastfeeding is feeding of an infant with only breast milk and no additional food, water, or other liquids (with the exception of medicines and vitamins, if needed) during the first six months of life. Infants who are exclusively breastfeed have less chance of becoming ill or dying from diarrhea, pneumonia, meningitis, ear infections and other infections [3]. Exclusive breastfeeding is the most widely known and effective intervention for preventing early-childhood deaths. Optimum breastfeeding practices can prevent 1.4 million deaths worldwide among children under five every year, despite this the prevalence of exclusive breastfeeding (EBF), in many developing countries including Ethiopia is low during the first six months of life [4].

Globally, pre-lacteal feeding is practiced in many countries; the highest prevalence in southeast and central Asia (54.6–93.9%) [2, 5–8] and at a modest rate in Latin America (20–45%) [9]. In Africa, most of the mothers (10.8–75.2%) offer prelacteal feeds to their newborn [5, 10–12]. Although 52% of Ethiopian newborns benefited from breastfeeding within one hour of birth, overall, nearly three children in every ten (27%) are given pre-lacteal feeds within the first three days of life [13].

In Ethiopia a high prevalence of prelacteal feeding practices have been reported in different part of the country; Harari, (45.4%) [14], Jimma, (17%), Sidama, (40.8%), and West Gojam (48.3%) [15–19].

Every day, three to four thousand infants died in developing world from diarrhea and acute respiratory infections. Even though pre lacteal feeding is not the direct cause of this death, it is one of the contributing causes of death as comorbidity with diarrhea and respiratory tract infection [14]. Pre-lacteal feeds increases the risk of illnesses such as diarrhea and other infections and allergies, particularly if they are given before the baby has had colostrum. Pre-lacteal feeds affect stimulation of breast milk production, suckling and mother-baby bonding [20]. Children who exposed for pre-lacteal feeding before six months of age were 16 times more likely to develop diarrhea or pneumonia [21].

Most mothers practice pre-lacteal feeding because they believe that; it gives laxative effect, clean meconium from the gut or has rehydration effect for newborns. But these prone the newborn to contamination and diarrhea [22]. Even though colostrum is three times richer in vitamin A and ten times richer in beta-carotene than mature milk), some believe pre lacteals are necessary substitutes for colostrum [1, 3].

The commonly used pre-lacteal feed practiced in Ethiopia includes; raw butter, plain water and milk-other than breast milk [23]. Ethiopia implements exclusive breastfeeding as one of the components of Primary Health Care and National Infant and Young Child Feeding (IYCF) Guideline, which discourage pre-lacteal feeding practices, but pre-lacteal feeding practice is still a neglected challenge [3]. Additionally predictor factors for pre-lacteal feeding were not well studied in Ethiopia particularly in the study area. Findings of this study will be used as baseline data, and hypothesis generation for researchers. It helps for policy makers and managers to develop appropriate plan and intervention program to reduce the morbidity and mortality rate of infant in Ethiopia. It also helps for Community health workers (health extension workers) and health care providers, for counseling and health education on pre-lacteal feeding practice and breast feeding. Therefore, the aim of this study was to assess Pre-lacteal feeding practice and its determinants among mothers of children age less than 24 months old in Soddo zuria district, Wolaita zone, southern Ethiopia.

## Methods

### Study area

The study was conducted in Sodo zuria district, wolaita Zone, one of the 13 Zones in the southern Nations Nationalities and People’s Region (SNNPR). Sodo zuria district is one of the district of Wolaita zones, which is located 327 km from Addis Ababa and 151 km far from Hawassa, capital city of SNNPR. The district is administratively structured by 36 kebele’s (the smallest administrative unit in Ethiopia) and has a total population of 176,810 of which 86,637 are males and 90,173 are females. About 9159 of the total population have children less than two years of age (https://en.wikipedia.org/wiki/Sodo_Zuria).

### Study design and period

Community-based cross-sectional study was conducted from February 15 to March 12, 2017.

## Population

### Source population

All Mothers of children less than 24 months of age in Sodo zuria district.

### Study population

Randomly selected Mothers of children less than 24 months of age in Sodo zuria district.

## Eligibility criteria

### Inclusion criteria

All Mothers of children less than 24 months of age who were residents of Sodo zuria district for six month and above were included in the study.

### Exclusion criteria

Mothers who were seriously ill and unable to communicate were excluded from the study.

### Sample size determination

Sample size was determined based on the formula used to estimate a single population proportion.

Estimated proportion of pre-lacteal feeding (28.92%) was taken *from* the previous study conducted in Ethiopia [23].

Then since we used multistage sampling, we consider design effect of 1.5, then 316 × 1.5 (design effect) = 474 and by adding 10% non-response rate the final sample size was 521mothers having children less than twenty-four months old.

### Sampling technique and procedure

Multi-stage sampling technique was used to recruit study participants. Sodo zuria district has a total of 36 kebeles, among these, 6 kebeles were selected by lottery method. The required sample size from this, six kebeles was selected by proportional allocation to size. By using Health extension workers family record book as a sampling frame systematic random sampling technique was used to select study participants. Sampling interval, K was calculated (by dividing total number of sample from each kebele to the study subjects in each kebele, which was found to be 3). The starting household was identified by lottery method and every 3rd household mother from each Kebeles was recruited for the study until the required sample size fulfilled.

### Method of data collection

The data collection tool was semi structured questionnaire which was adapted and modified from Ethiopian Demographic Health Survey and the national nutrition survey questioner. The data was collected through face to face interview using semi structured questionnaire.

Data collection was done by six trained BSc. holder nurses and two MSc. holder nurses supervised the data collection.

### Study variables

#### Dependent variable

Pre-lacteal feeding.

#### Independent variables

Maternal demographic factors, Child age, birth order, maternal health related factors, maternal knowledge, Colostrum’s avoidance, Breast feeding initiation, Families/other persons’ influence.

### Operational definition

*Pre-lacteal feeding*; giving of fluid or semisolid food before breast feeding to an infant during the first three days after birth [1].

*Colostrum’s avoidance*; includes; pumping and discarding colostrum during the first five days after birth [24].

*Extended family*: is family includes colleagues, aunts or uncles and grandparents living together, *Single parent family*: is only mother with her children with no father,

*Nuclear family*: is family type consists of two parents and children.

### Data quality and control

The questionnaire was prepared in English and translated to Wolatia language and back translated to English by two language experts to check for consistency of the questionnaire.

Training was given for data collectors and supervisors for three days and mock interview was practiced during the training to ensure the clarity and consistency questioners.

The pre-testing was conducted in 5% of the sample size of mother in similar area which is not selected in study area. For this purpose, Kokate kebele, which is one of the kebeles of the district and is not included as study kebeles, is selected to establish accuracy of questions and clarity and to determine the length of interviews. During pre-testing effort was made to check for consistency in the interpretation of questions and to identify ambiguous items. After review of the instruments all suggested revisions were made before being administered in the actual study. Its completeness and errors on spot and during data collection was cleaned and Coded before data entry and there were daily meeting at the end of data collection to identify any error and challenge.

### Data processing and analysis

The data was cleaned manually, coded and entered into Epi Data version 3.02 and was exported to SPSS version 22 software for analysis. After coding, and entering the data to the software descriptive statistics were used to calculate the result in proportion, frequencies, cross tabulation, and measure of central tendency. Tables and graphs were used to present the result. A bivariate binary logistic regression was used to identify candidate variables for the final model (multivariate binary logistic regressions) at *p*- value < 0.20. Finally, the independent predictors or variables which had significant association were identified by using multivariate binary logistic regressions. The cut point to declare the presence of an association between the dependent and independent variable was *p*-value < 0.05 with 95% confidence interval.

## Results

### Socio demographic characteristics

Five-hundred twenty one mothers having children less than 24 months of age were drawn and 495 consented to participate and included in this study resulting a response rate of (95.01%). The mean ages of respondents were 31.48 years (± SD 6.77) with age range from 18 to 46 years. Out of the total respondents 272 (57.0%) were with age range of 26–35 years. Majority of the mothers; 439(88.7%) had nuclear type of families and the rest 28(5.7%), 19(3.8%) were extended and single parent families respectively. One-hundred ninety seven (39.8%) were unable to read and write. More than two-third of the respondents (68.3%) were Protestant religion followers and 329 (66.5%) were house wives followed by merchants 127 (25. 7%). The mean age of children was 13.65 with (SD ±6.388) months.

### Health care service utilization of mothers

Regarding maternal health service utilization, of the total respondents 456 (92.1%) mothers were attending ANC; 129 (26.1%) utilized ANC four times and above, 435 (95.4%) had got breastfeeding counseling at ANC Clinic. More than three-fourth (79%) delivered their child at government health facility. The mode of delivery of majority of the participants (90.7%) was spontaneous vaginal delivery and health professionals assisted their delivery.

### Prevalence of pre-lacteal feeding practices

Out of 495 who had ever breastfed their index child; 102 (20.6%) (95% CI; 17.5, 24.4) were reported giving pre-lacteal feeds to their infants within the first three days before giving breast milk. The most common types of pre-lacteal feeding were plain water; 38 (7.7%) followed by butter; 23 (4.6%). The major reason for PLF were; mothers believed that breast feed only does not satisfy the new born; 32 (6.5%), cultural practice; 25 (5.1%), to clean infant’s bowel throat /Mouth; 21 (4.2%) (Table 1).

**Table 1:** Pre-lacteal feeding practices among mothers having children less than 24 months of age in Sodo zuria district, southern Ethiopia, 2017 [*n* = 495]

Variables	Category	Frequency	Percentages
Pre lacteal food given for the Child	Yes	102	20.6
	No	393	79.4
Types of pre lacteal food	Plain water	38	7.7
	Cow milk	18	3.6
	Water and “‘natra”’	14	2.8
	Butter	23	4.6
	Others	9	1.8
Reasons for pre lacteal feeding	Breastfed only is not satisfy the new born	32	6.5
	To clean infant’s bowel throat/Mouth	21	4.2
	Maternal medical illness	8	1.6
	cultural practice	25	5.1
	To calm/soothe the baby	11	2.2
	Others	5	1.0

### Source of information to provide pre lacteal feeding

**Fig. 1:**
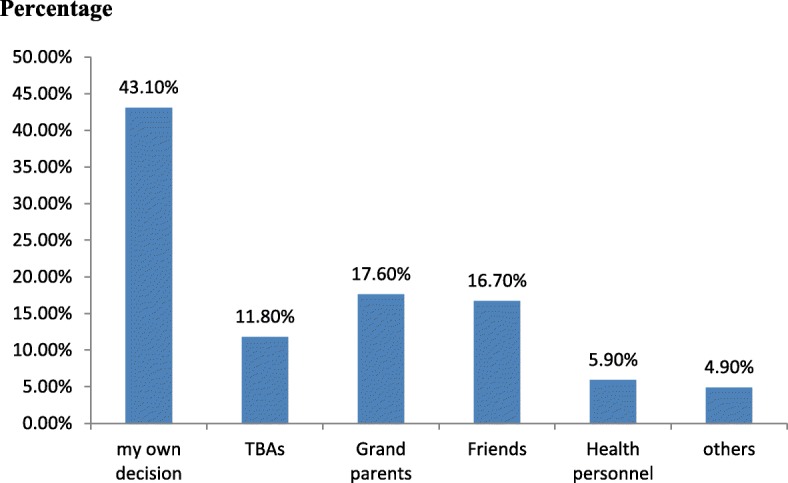
Source of information to provide pre lacteal feeding among mothers of children age less than 24 months old in Sodo zuria district, Wolaita zone, southern Ethiopia, 2017

Majority of the respondents (43.1%) provide pre lacteal feeding for their newborn with their own decision, and 17.6% of respondents provide pre lacteal feeding due to grandparents’ advice (Fig. 1).

### Colostrum avoidance

Of the total respondents; 453 (91.5%) feed colostrum for their infants within the first five days after delivery and 42(8.5%) had ‘squeeze & discarded’ colostrum. Out of those who gave colostrum for their infants, 241(48.7%) initiated breastfeeding within one hour, 170 (34.3%) were initiated after more than an hour. The main reasons for colostrum avoidance were maternal medical illness 20(4%), and insufficient breast milk 9 (1.8%) (Table 2).

**Table 2:** Distribution of colostrum feeding practice and initiation of breast feeding among mothers of children less than 24 months of age in Sodo zuria district, 2017 [*n* = 495]

Variables	Categories	Frequency	Percentage
Mothers avoid colostrum	Yes	42	8.5
	No	453	91.5
Time of initiation of breast feeding (*n* = 453)	< 1 h	241	48.7
	1-6 h	170	34.3
	7-12 h	14	2.8
	> 12 h	28	5.7
Reasons for colostrum avoidance (*n* = 42)	Maternal medical illness	20	47.6
	for the child growth	7	16.6
	Breast has no milk	9	21.4
	Cause Abdominal discomfort and diarrhea	6	14.2

### Knowledge on pre-lacteal feeding

Majority of the respondents 409 (82.6%) knew about the disadvantages of pre lacteal feeding and the rest 86 (17.4%) mothers did not know the risks of pre-lacteal feeding (Table 3).

**Table 3:** Knowledge on pre-lacteal feeding among mothers having children less than 24 months of age in Sodo zuria district, southern Ethiopia, 2017[*n* = 495]

Variables	Categories	Frequency	Percentage (%)
Knows risk of pre lacteal feeding	Yes	409	82.6
	No	86	17.4
Information on problems of pre lacteal feeding	Poor growth	74	14.9
	Vomiting	107	21.6
	Diarrhea	126	25.5
	Infection	102	20.6

### Factors associated with pre-lacteal feeding practice

During bivariate logistic regression analysis; family type, delivery attendants, breast feeding counseling; knowledge about risks of pre-lacteal feeding and avoidance of the colostrum were statistically associated with pre-lacteal feeding. Variables which showed significant association on bivariate analysis were entered to multivariate logistic regression analysis to identify independent predictors of pre lacteal feeding.

In multivariable logistic regression analysis mothers living with extended family type, lack of breastfeeding counseling, and avoidance of colostrum were statistically significant positive predictors of pre-lacteal feeding practice. Mothers who live with extended family type were more than 10-fold likely to give pre-lacteal feeding as compared to those mothers who live with nuclear family type. (AOR = 10.65, 95% CI: 1.05, 10.71). Mothers who didn’t get breastfeeding counseling were about 5 times more likely to provide pre-lacteal feeding as compared to those mothers who got breast feeding counseling (AOR = 5.164, 95% CI: 1.763,15.127). Mothers who avoided colostrum were nearly ten times more likely to provide pre-lacteal feeding as compared to those mothers who fed colostrum to their infants (Table 4).

**Table 4:** Factors associated with pre-lacteal feeding practices among mothers having children less than 24 months of age in Sodo Zuria district, southern Ethiopia, 2017[*n* = 495]

Variables	Pre-lacteal feeding given		COR (95% CI)	AOR (95% CI)
	Yes	No		
Family type				
Nuclear family	366 (83.37%)	73 (16.63%)	1.00	1.00
Single parent family	13 (68.4%)	6 (31.6%)	1.615 (0.255,10.22)6)	0.513 (0.044,5.977)
Extended family	21 (75%)	7 (25%)	10.5 (1.754,62.839)	10.644 (1.05,10.713)*
Others	2 (22.2%)	7 (77.8)	0.698 (0.14,3.428)	1.293 (0.144,11.604)
Counseling on Breastfeeding				
Yes	73 (16.7%)	362 (83.3%)	1.00	1.00
No	12 (57%)	9 (43%)	6.612 (2.688,16.26)	5.164 (1.763,15.127)*
Place of delivery				
Governmental Health facility	67 (17.1%)	324 (82.9%)	1.00	1.00
At home	35 (33.6%)	69 (66.4%)	.408 (0.251, 0.662)	.747 (.342,1.631)
Person delivered				
Health professionals	73 (17.4%)	346 (82.6%)	1.00	1.00
TBAs	29 (38.15%)	47 (61.85%)	.342 (.202,.579)	.871 (.331,2.291)
Mothers knows the risk of Pre lacteal feeding				
Yes	60 (15.2%)	333 (84.8%)	1	1
No	26 (25.4%)	76 (74.6%)	.527 (.312,.889)	.664 (.281,1.569)
Mother Avoids colostrum				
Yes	75 (21.2%)	378 (78.8%)	1.00	1.00
No	27 (62.7%)	15 (37.3%)	9.072 (4.605,17.872)	9.715 (3.457,27.303)*

*Significant at *p*-value ≤ 0.05

## Discussion

The study showed that exclusive breastfeeding practices were sub-optimal in the study area due to wide spread introduction of Pre-lacteal feeding. The prevalence of pre-lacteal feeding in the study area was found to be (20.6%). This finding is higher when compared with national prevalence of prelacteal feeding; from the total sample of children (8%) were fed pre-lacteals [25]. And it’s consistent with studies done in Nepal (23.1%), and the Upper East Region of Ghana (18%) [2, 26]. The current prevalence of pre-lacteal feeding is higher than a study done in Benin City, Nigeria (11.7%), and Ethiopian studies conducted in Arbaminch zuria district (8.9%), Enderta district (12.8%),Jimma (12.6%) [12, 15–17]. This variation might be due to the difference in culture, the population character, and geographic distribution. On the other hand, prevalence of prelacteal feeding in this study was so much lower than studies done in Vietnam (74.7%), India (45.4%), Harari, Eastern Ethiopia and (48.3%) West Gojam [7, 15, 19, 27]. This might be due to the difference in level of awareness of involved population about the health impact of prelacteal feeding, health service access for ANC follow up and counseling, religious, and cultural acceptance of prelacteal feeding like butter.

The major reason for PLF were; mothers believed that breast milk only does not satisfy the newborn (6.5%), cultural practice (5.1%), misperception of mothers that they provide PLF to clean infant’s bowel, throat or mouth (4.2%). The major influence to provide the PLF were; mothers own decision; 44(8.9%), Grandparents advise; 18(3.6%) followed by advice from friends; 17 (3. 4%). The study further revealed that major reasons for the pre-lacteal feeding were the insufficient milk/ delayed lactation (31.25%), elder’s advice (29%) and family custom (25%). This finding is consistent with another study conducted in Nigeria [10, 15] which was found (51.1%) and (62.9%) mothers giving pre-lacteal feeds due to insufficient milk respectively.

Those mothers who live with extended family type were 10 times more likely to give pre-lacteal feeding as compared to those mothers who live with nuclear family type. This is in line with a study conducted in India which reported that Nineteen (61.3%) mothers out of 31 practiced prelacteal feeding among extended family, 38 (42.7%) out of 89 given pre-lacteal feeding as compared to the 7 (23.3%) out of 30 amongst the nuclear family practiced pre-lacteal feeds. Respondents from extended family are more likely to practice pre-lacteal feeding due to the family customs [26].

Mothers who didn’t get breast feeding counseling were five times more likely to practice pre-lacteal feeding when compared to those mothers who got breast feeding counseling. a Study done in Maharashtra, India is consistent with this finding [26]. This might be due to inadequate training regarding breastfeeding counseling, or due to knowledge gap about the importance of colstrom and health impact of pre lacteal feeding, lack of ANC access.

Mothers who avoided colostrum were 10 times more likely to give pre-lacteal feeding as compared to those mothers who fed colostrum to their infants. This is consistent with qualitative study conducted in the Raya kobo district, Amhara region showed that colostrum is thought to cause abdominal cramps and raw butter is thought to clean infants’ stomachs. Therefore, untrained traditional birth attendants advised mothers to discard colostrum and feed their infants with raw butter before breastfeeding initiated [11]. Moreover this finding is consistent with community based study done in Ethiopia which showed that mothers who discard colostrum were almost nine times more likely to practice pre-lacteal feeding [28].

## Conclusion

Pre-lacteal feeding is commonly practiced among mothers of children less than two years of age in Sodo zuria district. This makes breastfeeding practices sub-optimal in the study area. The most common types of pre-lacteal feeding were plain water; 38 (7.7%) followed by raw butter; 23 (4. 6%). The major reason for PLF were; mothers believed that breast feeding only does not satisfy the newborn, cultural practice, to clean infant’s bowel throat/mouth and mothers provide pre lacteal feeding with their own decision. Lack of breastfeeding counseling, living with extended family type and discarding of colostrum were statistically significant positive predictors of pre-lacteal feeding practice. Therefore, attention has to be given on breastfeeding counseling during ANC follow up with special focus on mothers who live with extended family and Health education about the importance of colostrum and health impact of pre lacteal feeding.

## Abbreviations

ANC: Anti natal care
AOR: Adjusted odd ratio
CI: Confidence Interval
EBF: exclusive breastfeeding
EDHS: Ethiopian Demographic Health Survey
IYCF: Infant and Young Child Feeding
PLF: Pre lacteal feeding
SD: Standard Deviation
SNNPR: South Nation Nationalities and peoples region
SPSS: Statistical Package for Social Sciences
